# Electrochemistry in a Two- or Three-Electrode Configuration to Understand Monopolar or Bipolar Configurations of Platinum Bionic Implants

**DOI:** 10.3390/mi14040722

**Published:** 2023-03-24

**Authors:** Alexander R. Harris, David B. Grayden, Sam E. John

**Affiliations:** 1 Department of Biomedical Engineering, University of Melbourne, Melbourne 3010, Australia; 2 Graeme Clark Institute, University of Melbourne, Melbourne 3010, Australia

**Keywords:** in vivo electrochemistry, electrode configuration, two-electrode, three-electrode, electrochemical methods

## Abstract

Electrodes are used in vivo for chemical sensing, electrophysiological recording, and stimulation of tissue. The electrode configuration used in vivo is often optimised for a specific anatomy and biological or clinical outcomes, not electrochemical performance. Electrode materials and geometries are constrained by biostability and biocompatibility issues and may be required to function clinically for decades. We performed benchtop electrochemistry, with changes in reference electrode, smaller counter-electrode sizes, and three- or two-electrode configurations. We detail the effects different electrode configurations have on typical electroanalytical techniques used on implanted electrodes. Changes in reference electrode required correction by application of an offset potential. In a two-electrode configuration with similar working and reference/counter-electrode sizes, the electrochemical response was dictated by the rate-limiting charge transfer step at either electrode. This could invalidate calibration curves, standard analytical methods, and equations, and prevent use of commercial simulation software. We provide methods for determining if an electrode configuration is affecting the in vivo electrochemical response. We recommend sufficient details be provided in experimental sections on electronics, electrode configuration, and their calibration to justify results and discussion. In conclusion, the experimental limitations of performing in vivo electrochemistry may dictate what types of measurements and analyses are possible, such as obtaining relative rather than absolute measurements.

## 1. Introduction

The field of bionics places electrodes in vivo for electrochemical sensing, electrophysiological recording, and electrical stimulation of tissue. Their use ranges from acute and chronic experiments in animal models to permanent implantation in patients for clinical applications. They may be placed in or on various organs or tissue, including the central and peripheral nervous system, heart, cochlea, retina, blood vessels, and muscle. Common applications of electrodes are: recording electrophysiological function of neural or muscle tissue to understand or detect disease [1,2] and control prosthetic devices [3]; stimulation of neural tissue to provide sensory input such as the cochlear implant [4]; control disease symptoms such as tremor and rigidity for Parkinson’s disease [5]; or detection of neurotransmitters such as dopamine within the brain of animal models [6].

Electrodes used in vivo have a range of geometries and materials, including carbon microfibres, micro- and macro-sized planar platinum films, platinum bands and cones. Typical electrochemical techniques applied to these electrodes include fast scan cyclic voltammetry and chronoamperometry for chemical sensing, high-pass-filtered open circuit potential measurements for electrophysiological recording, chronopotentiometric voltage transients for tissue stimulation, and cyclic voltammetry and electrochemical impedance spectroscopy (EIS) for electrode/tissue interrogation. For example, the conical Utah electrode array has been implanted in rat, feline and Rhesus macaques for up to a year, with regular measurement of EIS at 1 kHz and charge storage capacity measured from cyclic voltammetry [7,8,9,10]. The responses were highly variable over time, with authors speculating about the causes including biofouling, movement of encapsulating tissue, insulation failure or electrode failure.

In a more recent example [11], a cochlear implant underwent a series of electrochemical analyses in an in vitro three-electrode configuration using an Ag|AgCl reference electrode and large platinum foil counter electrode prior to implantation in a rat model; in vivo electrochemistry was then performed in a bipolar or tripolar electrode configuration with the centre electrode acting as the working electrode, and, presumably, the counter and reference leads shorted to the other adjacent electrodes of equivalent size. The authors speculated that implantation and formation of a tissue capsule around the implant increased changes in potential during voltage transients and the access voltage. However, no control experiments were performed to assess changes in response with the different in vitro and in vivo electrode configuration or reference electrode potential.

In a series of other recent studies, we have investigated the impact of implantation on electrochemical response; it was shown that electrodes are activated during current passage, significantly altering their electrochemical behaviour [12]. Proteins also adsorb onto the electrode surfaces, partially blocking the electrode [13]. It was found that bone formation between electrodes could affect electrochemical response, but scar tissue formation between electrodes was no different on electrochemical response compared to other tissue types [12]. We have compared the in vitro and in vivo electrochemistry, we demonstrated that solution composition affects the electrochemical response, and the current, the best in vitro model of in vivo electrochemistry for testing bionic electrodes is degassed saline [12]. We did note that different reference electrodes were used and this could affect the electrochemical response. In vivo electrochemistry uses a variety of different electrode configurations that are not typical of benchtop electrochemistry, including different reference electrodes and two- or three-electrode configurations in the same experiment. While benchtop electrochemistry is commonly performed in a three-electrode configuration with a significantly larger counter electrode compared to the working electrode, in vivo electrochemistry often uses counter and working electrodes of similar size, or a two-electrode system with similar sized electrodes. Yet, the impact of electrode configuration on in vivo electrochemical response is rarely acknowledged.

Issues around electrode configuration for biosensing are often dismissed, as they draw low current, do not require long-term stability, and are cheaper to run in a two-electrode configuration [14]. However, in one example, the effect of electrode configuration was assessed on DNA hybridization on the electrode surface, which found that a three-electrode system improved the stability of impedance measurements [15]. In addition, dopamine detection in a rat model in a three-electrode configuration was better able to compensate for changes in impedance compared to a two-electrode configuration [16].

Biosensors are increasingly being used for chronic measurements, with greater risk of electrode variation and degradation, which may affect electrochemical function [17]. When it comes to bionics applications, various electrode configurations are used to control current spread for tissue stimulation, but their impact on the electrochemical response has not been discussed [18]. Some impacts of using a two- or three-electrode configuration on electrochemical analysis of batteries have been discussed previously [19]. Even in the electrochemical literature, the discussion of electrode configuration is very limited [20]. Some technical notes available from potentiostat manufacturers mention potential drift (https://www.gamry.com/application-notes/instrumentation/two-three-four-electrode-experiments/#:~:text=Three%2Delectrode%20setups%20have%20a,occur%20at%20the%20counter%20electrode (accessed on 14 November 2022)) and that a counter electrode should be the same size or bigger than the working electrode so that it can provide a large capacitance charge, prevent solution contamination or kinetics limitations (https://www.basinc.com/products/ec/faqele#Three (accessed on 14 November 2022). https://www.palmsens.com/knowledgebase-article/electrodes-of-a-potentiostat/ (accessed on 14 November 2022)). So, while electrochemical experts may understand the theoretical impacts of changing electrode configuration, there are actually very few examples detailing the specific effects changes in electrode configuration have on electrochemical response.

Therefore, this article provides clear details about the impact that changes in electrode configuration have on the electrochemical measurements usually performed on implanted electrodes, to assist non-experts in electrochemistry. Experiments are performed on platinum electrodes, the material most commonly used for bionics applications. It presents simple methods for identifying when the electrode configuration is affecting the system, such as when the counter electrode is limiting the electron transfer kinetics and requires adjustment. This will provide guidance on performing in vivo electrochemical experimentation, ensuring the electrode configuration is not affecting the electrochemical analysis.

## 2. Experimental

**Figure 1:**
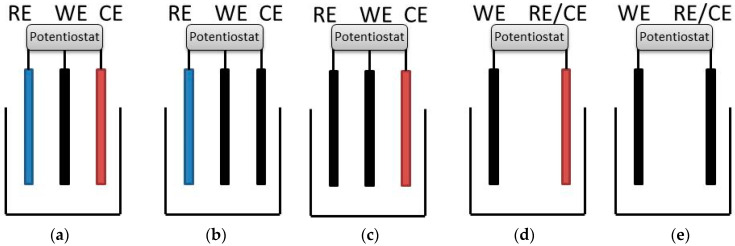
Varying electrode configurations used to assess impact on electrochemical response as defined in the experimental section. Black—0.6 mm diameter platinum disc, Red—1 cm^2^ platinum mesh, Blue—Ag|AgCl (3 M KCl).

Sodium chloride and hexaammineruthenium(III) chloride (Ru(NH_3_)_6_Cl_3_) were purchased from Sigma-Aldrich, Melbourne. The working electrode was a 0.6 mm diameter platinum disc (CH Instruments), a typical size of cochlear implant electrodes. Electrodes were tested in either three- or two-electrode configurations (Figure 1) using: (1) a Ag|AgCl (3 M KCl) reference electrode and a 1 cm^2^ platinum mesh counter electrode (significantly larger than the working electrode); (2) a Ag|AgCl (3 M KCl) reference electrode and a 0.6 mm diameter platinum disc counter electrode; (3) a 0.6 mm diameter platinum disc reference electrode and platinum mesh counter electrode; (4) a platinum mesh as a combined reference/counter electrode; or (5) a 0.6 mm diameter platinum disc as combined reference/counter electrode. Disc electrodes were polished with 0.3 μm alumina slurry on Microcloth polishing cloth (Buehler), rinsed in deionised water and gently dried (Kimwipe) before use. Experiments were performed with a 1010E potentiostat (Gamry, Warminster). Test solutions of 0.1 M NaCl were degassed with argon for at least 10 min before performing electrochemistry. PBS was not used to buffer the solution as phosphate adsorbs to platinum, altering its electrochemical response, and is a poor model of in vivo electrochemistry [12,21]. Removal of platinum oxide by performing cyclic voltammetry in concentrated H_2_SO_4_ solutions was not undertaken, as this acid polished platinum is a poor model of in vivo electrodes [21]. Faradaic response was measured by the addition of 5 mM Ru(NH_3_)_6_Cl_3_ to the test solution [22].

The open circuit potential was measured for 10 s, with a maximum change of 1 mV observed over this time period. Charge storage capacity measurements were performed on the second cycle of a voltammogram over a range of 0.8 to −0.8 V (unless stated otherwise) at a scan rate of 100 mV s^−1^. The cathodic charge storage capacity (CSCc) was calculated from the total cathodic current over the forward and backward scan; the anodic charge storage capacity (CSCa) was calculated on the backward scan from where the voltage crossed 0 A, up to 0.8 V. EIS was performed at 0 V vs. the reference electrode (unless described differently) with a 5 mV amplitude over a frequency range of 0.1 Hz to 1 MHz. Equivalent circuit fitting of the EIS data was performed with Gamry Echem Analyst 7.10.0. Average and standard deviations were calculated from three repeats on the same electrode freshly polished between experiments (Table 1 and Table 2). Chronopotentiometric voltage transients were not performed, as the Gamry potentiostat was unable to control or measure the initial potential.

## 3. Results

### 3.1. Cyclic Voltammetry of Platinum in Different Electrode Configurations

**Figure 2:**
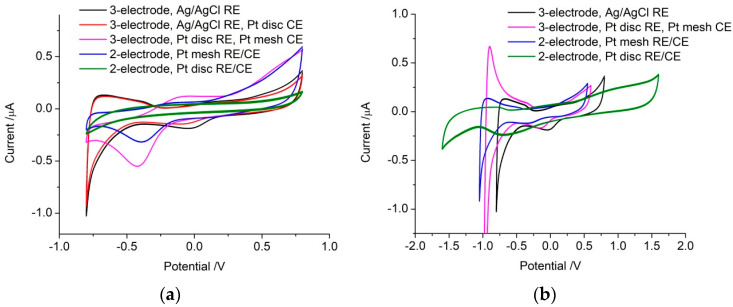
(**a**) Second voltammetric cycle of a platinum electrode in degassed 0.1 M NaCl from 0.8 to −0.8 V at a scan rate of 100 mV s^−1^ in a three- or two-electrode configuration. (**b**) Varying potential range to determine water window.

The electrochemical response was initially assessed in a typical three-electrode setup with a freshly polished platinum disc working electrode, an Ag|AgCl reference electrode and a large platinum mesh counter electrode. The open circuit potential was ~289 mV (Table 1). Voltammetry from 0.8 to −0.8 V was consistent with previous reports [21], showing a broad reduction peak around −65 mV due to platinum oxide reduction, increasing reduction current below −500 mV from hydrogen adsorption, and water reduction (Figure 2a). Switching the scan direction, a broad oxidation current extended to −270 mV from hydrogen desorption and increasing current around 800 mV from platinum oxide formation and water oxidation. The CSC was measured, giving a larger CSCc than CSCa, largely due to the reduction of platinum oxide (Table 1).

The counter electrode was then changed to a 0.6 mm diameter platinum disc, a similar size to the working electrode, while retaining the Ag|AgCl reference electrode. The open circuit potential was similar to the previous measurement at 236 mV (Table 1). Voltammetry was almost identical in shape, with a slight decrease in current magnitude, resulting in a decrease in both CSCc and CSCa (Figure 2a).

The reference electrode was then changed to a freshly polished platinum disc while the large platinum mesh was used as the counter electrode again. The open circuit potential now shifted substantially to 0 V (Table 1). The voltammetry appeared shifted to more negative potentials, with a much larger platinum oxide reduction peak around −420 mV, no visible hydrogen adsorption or desorption, and platinum oxide formation occurring above 290 mV (Figure 2a). The CSCc and CSCa over this potential range were substantially larger than the three-electrode configuration using an Ag|AgCl reference electrode. By adjusting the potential window 200 mV more negatively, from 0.6 V to −1 V, the voltammetric response became very similar to the three-electrode configuration using an Ag|AgCl reference electrode (Figure 2b).

The reference and counter electrode leads were then connected together to the large platinum mesh electrode in a two-electrode configuration with the platinum disc working electrode. The open circuit potential at −38 mV was similar to the previous response using the platinum disc as a reference electrode (Table 1). The voltammetry was also very similar to the previous response, particularly when the potential window was shifted more negative (Figure 2).

Finally, the reference and counter electrode leads were connected together to a platinum disc electrode in a two-electrode configuration with another platinum disc as working electrode. The open circuit potential was similar at 19 mV (Table 1). However, the voltammetry was featureless over the 0.8 to −0.8 V range, with a significantly smaller CSCc and CSCa (Figure 2). Adjusting the potential range, this electrode configuration allowed a safe potential window from approximately 1.6 to −1.6 V with a broad platinum oxide reduction peak around −670 mV and platinum oxide formation occurring above 450 mV (Figure 2b). Over this wider potential range, CSCc was 4.8 µC and CSCa was 3.7 µC.

### 3.2. Electrochemical Impedance Spectroscopy of Platinum in Different Electrode Configurations

**Figure 3:**
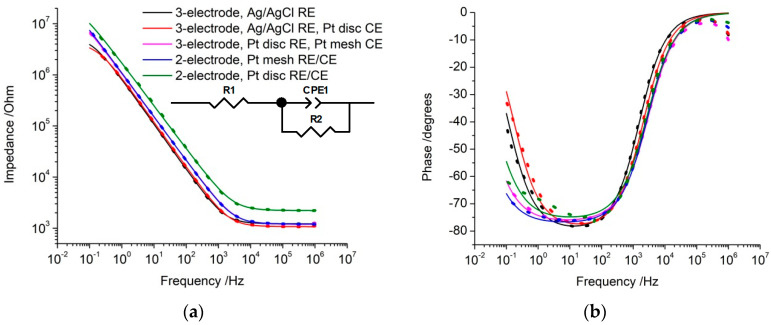
Electrochemical impedance of a platinum electrode in degassed 0.1 M NaCl at 0 V with an ac amplitude of 5 mV in a three- or two-electrode configuration. Equivalent circuit used to model the impedance is shown.

EIS was performed at 0 V vs. the reference electrode rather than at open circuit potential, as 0 V is well-defined and the open circuit potential can vary with conditions. The response in all electrode configurations was similar to previous reports of platinum, displaying a single time constant (Figure 3) [23,24]. The impedance at low frequencies is dependent on electrode properties, including area, and is correlated with signal-to-noise ratio of neural recording [25,26]. The total impedance at 10 Hz increased slightly when moving from an Ag|AgCl reference electrode to a platinum reference electrode and increased substantially when using a two-electrode configuration with a disc electrode for both working and counter/reference electrodes (Table 2).

The EIS response was fit with an equivalent circuit comprising resistors (R1 and R2) and a constant phase element (CPE1). R1 models the solution resistance, while the electrode/solution interface is modelled as a parallel constant phase element (CPE1) and polarisation resistance (R2). The constant phase element was used instead of a capacitor due to surface roughness or inhomogeneity in current distribution at the electrode surface. The significance of fitting this equivalent circuit was high, with a χ^2^ of less than 2 × 10^−3^ in all electrode configurations. Values for the fitted resistance, admittance (*Q*_0_), and power (*n*) terms for the constant phase element are listed in Table 2.

The solution resistance was little affected by the electrode configuration, but approximately doubled with a two-electrode configuration using a disc electrode for both working and counter/reference electrodes (Table 2). The admittance decreased slightly when moving from an Ag|AgCl reference electrode to a platinum reference electrode, and decreased substantially when using a two-electrode configuration with a disc electrode for both working and counter/reference electrodes. The polarisation resistance increased substantially in all configurations with a platinum reference electrode.

The change in EIS response when using a two-electrode configuration comprising a disc electrode for both working and counter/reference electrodes suggests the electrode-solution interface at the counter/reference electrode has affected the electrochemical response. Therefore, a second equivalent circuit was applied with the counter/reference electrode modelled as a second parallel constant phase element (CPE2) and polarisation resistance (R3) placed in series with the previous circuit. Compared to the previous model, this new fit had no impact on the solution resistance or any of the power terms, but both CPE1 and CPE2 increased, and R2 and R3 decreased. However, with so many model parameters, there was considerable freedom of movement in fitting CPE1, CPE2, R2, and R3. Furthermore, the CPE1/R2 elements and CPE2/R3 elements could be assigned to either working or counter/reference electrode. Therefore, the more complex equivalent circuit supports the fact that the electrode-solution interface at the counter/reference electrode is affecting the EIS response, but did not allow obtainment of accurate fitting parameters. Therefore, this more complex equivalent circuit was not used.

To determine the impact of changing electrode potential, the EIS was measured at −200 mV with a platinum disc reference electrode and platinum mesh counter electrode. Compared to 0 V, the impedance at 10 Hz decreased by 20 kOhm (15%), the solution resistance was unaffected, admittance increased by 9.2 × 10^−8^ S s^1/2^ (43%), *n* was unaffected, and polarisation resistance decreased to 6.5 MOhm. This response was almost identical to measurements at 0 V vs. an Ag/AgCl reference electrode. In a two-electrode configuration with a platinum mesh counter/reference electrode, EIS at −200 mV reduced the impedance at 10 Hz by 30 kOhm, solution resistance and *n* were unaffected, admittance increased to 24.5 × 10^−8^ S s^1/2^, and polarisation resistance decreased to 5.5 MOhm.

### 3.3. Voltammetry of Ru(NH_3_)_6_^3+^ in Different Electrode Configurations

The impact of electrode configuration on electrochemical sensing was assessed with the diffusion-controlled Faradaic reaction of Ru(NH_3_)_6_^3+^, which undergoes a reversible one-electron reduction:

Ru(NH_3_)_6_^3+^ + *e*^−^ ⇌ Ru(NH_3_)_6_^2+^

At fast scan rates, a peak-shaped voltammetric response is seen with a peak current given by

(1) $$i_{p}=(2.69\times 10^{5})n^{3/2}AD^{1/2}C\upsilon^{1/2}$$

where *n* is the number of electrons transferred, *D* is the diffusion coefficient (9.0 × 10^−6^ cm^2^ s^−1^) [27], *C* is the concentration, and *ν* is the scan rate.

**Figure 4:**
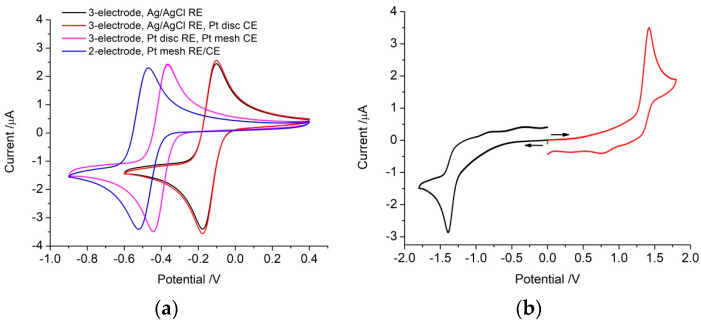

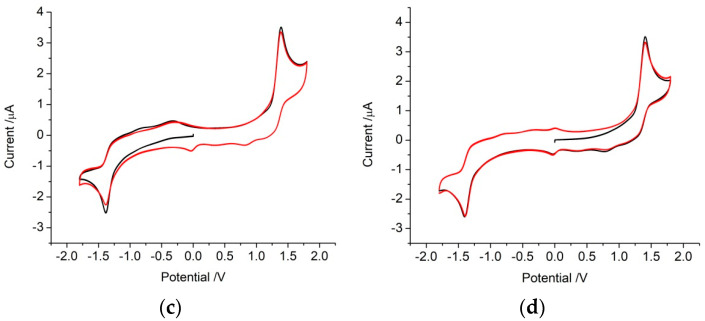
Cyclic voltammetry of a platinum electrode in degassed 0.1 M NaCl with 5 mM Ru(NH_3_)_6_^3+^ at a scan rate of 100 mV s^−1^ in a (**a**) three- or two-electrode configuration, (**b**–**d**) two-electrode configuration with a platinum disc reference/counter electrode over different potential ranges and directions, (**b**) starting from 0 V scanning either positive or negative, (**c**) two cycles scanning negatively, (**d**) two cycles scanning positively.

In a three-electrode configuration with an Ag|AgCl reference electrode and platinum mesh counter electrode, the reduction peak (*E*_pc_) was −175 mV, oxidation peak (*E*_pa_) was −102 mV, giving *E*^1/2^ of −139 mV and peak splitting (Δ*E*_p_) of 73 mV (Figure 4a). The cathodic peak current (*i*_pc_) was 3.33 μA and the oxidation peak current (*i*_pa_) 3.25 μA. Swapping to the platinum disc counter electrode had no noticeable impact on the Ru(NH_3_)_6_^3+^ voltammetry. With a platinum disc reference electrode and platinum mesh counter electrode, *E*_pc_ shifted to −442 mV, and *E*_pa_ to −365 mV, giving *E*^1/2^ −404 mV while Δ*E*_p_ was practically identical at 77 mV and no change in peak current was seen. This change in *E*^1/2^ is similar to the change in open circuit potential measured with the different reference electrodes (Table 1).

In a two-electrode configuration with a platinum mesh as counter/reference electrode, *E*_pc_ = −521 mV, *E*_pa_ = −470 mV, giving *E*^1/2^ = −496 mV and Δ*E*_p_ = 51 mV, with no change in peak height. However, the Faradaic response shifted to more negative potentials with each subsequent voltammetric cycle.

Finally, in a two-electrode configuration with a disc electrode for both working and counter/reference electrodes, a significant change in response occurred (Figure 4b–d). Scanning in a negative direction from 0 V to −1.8 V, a reduction peak appeared at −1392 mV with a peak current of 1.54 μA; no oxidation process was seen on the reverse scan (Figure 4b). On subsequent cycles, the reduction peak potential was unchanged but the peak current increased slightly. Scanning in a positive direction from 0 V to 1.8 V, an oxidation peak appeared at 1421 mV with a peak current of 2.51 μA, but no reduction peak was seen in the reverse scan. This oxidation current appeared despite there being no prior reduction of Ru(NH_3_)_6_^3+^. Once again, on subsequent cycles, the peak potential was unchanged but the peak current increased slightly. The voltammetric scans in the negative and positive directions appear as mirror images.

Scanning over the entire range from 0 V to −1.8 V and up to 1.8 V, the reduction peak occurred at −1384 mV and the oxidation peak at 1389 mV (Figure 4c). On the first cycle, the reduction peak current was 1.34 μA and the oxidation peak current was 2.33 μA. On the second cycle, the peak potentials were unchanged, but the reduction peak current decreased to 0.87 μA while the oxidation peak current was 2.00 μA. The peak currents were unchanged on further voltammetric cycles. Scanning from 0 V to 1.8 V then down to −1.8 V gave a similar response (Figure 4d). The oxidation peak was at 1406 mV and 2.35 μA and the reduction peak was at −1403 mV and 1.32 μA. Once again, this oxidation peak appeared despite no prior reduction of Ru(NH_3_)_6_^3+^. On the second cycle, the peak potentials remained the same, with the oxidation peak decreasing to 2.03 μA while the reduction peak remained at 1.33 μA.

## 4. Discussion

Based on the above results, a discussion of the impact different electrode configurations have on electrochemical response and, subsequently, the implications for in vivo electrochemistry will be presented.

### 4.1. The Impact of Varying Reference Electrode on Electrochemical Behaviour

The potential of the working electrode is controlled against the reference electrode. A small amount of current must flow through the reference electrode for it to function, but the majority of the current passes through the working and counter electrodes. When using the Ag|AgCl (3M KCl) reference electrode, the potential of the working electrode was well defined. Use of a stable, standardised reference electrode allows comparison across experiments and to other electrochemical systems. While all standard potentials are defined versus the standard hydrogen electrode (SHE), this requires hydrogen gas and a highly acidic solution, which is difficult to use. Therefore, the simpler Ag|AgCl or saturated calomel (SCE) reference electrodes are more commonly used. The potential of the Ag|AgCl electrode depends on the activity of Cl^−^ in the surrounding solution (other ions/chemical species, temperature, and pressure also affect the potential). The internal filling solution of the Ag|AgCl electrode (typically 3M or saturated KCl) is usually separated from the test solution with a porous glass frit that allows current to pass, but limits transport of Cl^−^, reducing any potential drift. Ag|AgCl coated with nafion was used to reduce potential drift when measuring in vivo dopamine concentrations, but the coating failed after 28 days [28]. Calibration of potential differences between reference electrodes can be undertaken by various measurements (e.g., open circuit potential or the *E*^1/2^ of a Faradaic reaction, such as the reduction of Ru(NH_3_)_6_^3+^).

A resistance between the reference and working electrodes will result in a potential loss, so the working electrode does not achieve the applied potential. This can be caused by blocking of the electrode surfaces or low solution conductivity, which can be mitigated by raising the solution conductivity or reducing the distance between the working and reference electrodes. The potential of a reference electrode can be further affected by a junction potential formed between two different solutions with different compositions (e.g., differing salt concentrations or aqueous/organic solvents). It can also be affected by a Donnan potential, where different charged species (e.g., proteins) are unable to cross the frit, causing a charge separation. Proteins that adsorb onto the reference electrode can remain permanently attached, affecting its potential. For all these reasons, simply changing the reference electrode material or coating an Ag|AgCl electrode is unlikely to result in a long-term stable in vivo reference potential. Subsequently, electrode potentials are normally calibrated after exposure to adsorbing species and in relevant solutions, and regularly tested for further changes in potential.

When using platinum as a reference electrode, its potential is undefined and can vary with conditions. Comparison with other experiments and systems can only be achieved by direct measurement of their relative potentials. However, the potential of the platinum electrode is highly dependent on its surface state (level of oxide, hydride or adsorption of other chemical species) and solution composition. This can lead to large changes in electrode potential between or even within experiments.

When assessing electrodes for bionics applications, it is common to define a safe potential range between water oxidation and reduction reactions, which was ~0.8 to −0.8 V vs. Ag|AgCl (3M KCl). Subsequently, when performing electrical stimulation of tissue using voltage transients, the maximum safe current amplitude is defined by this water oxidation and reduction potential. However, changing to a platinum quasi-reference electrode shifted the safe potential window in 0.1 M NaCl to ~0.6 to −1 V. By retaining the safe potential range as 0.8 to −0.8 V but using a platinum reference electrode, it is possible to unintentionally apply anodic current above the water electrolysis potential. This led to formation of platinum oxide and a large voltammetric peak associated with its subsequent reduction (Figure 2). Other Faradaic reactions also occur within the water window, including oxygen reduction or dopamine oxidation. The potential for these reactions will also shift with a different reference electrode (e.g., Figure 4). While Faradaic reactions are relatively easy to visualise during cyclic voltammetry (as peaks), they are harder to assess on a chronopotentiometric curve (as slight changes in gradient). The change in Faradaic reaction *E*^1/2^ with reference electrode must be taken into account when assessing voltage transients. Furthermore, changes in starting potential of a voltage pulse can impact on the safe stimulation current [29]. Ultimately, the undefined and variable potential of a platinum reference electrode when used in vivo limits the assignment of safe windows or Faradaic reaction potentials.

EIS can also be affected by changing the reference electrode. While solution resistance is independent of electrode potential, the capacitance (or admittance and polarisation resistance) are affected [30,31]. Furthermore, the response at different potentials can be significantly altered when charge transfer reactions occur [32]. At 0 V vs. Ag|AgCl, the platinum oxide reduction process was present (Figure 2), while at 0 V vs. the platinum reference electrode, the majority of charge was associated with capacitance. Measuring EIS at 0 V vs. the platinum reference electrode increased impedance at 10 Hz and the polarisation resistance while the admittance decreased (Table 2). EIS at −200 mV vs. the platinum reference electrode was equivalent to 0 V vs. the Ag|AgCl reference electrode. This variation of EIS response with potential has important ramifications for assessing electrodes in vivo, as demonstrated recently [12]. Baseline EIS responses may be obtained in vitro prior to implantation and then measured over time after implantation. Any change in reference electrode, solution composition, and protein adsorption after implantation will alter the reference electrode potential and subsequent EIS measurements. Typically, increases in impedance are blamed on the formation of scar tissue but this fails to account for other factors on the impedance response. The common measurement of impedance at 1 kHz has been shown to have limited utility in understanding electrode function [26]. However, a single measurement at 10 Hz, which is more dependent on electrode size, would still provide insufficient information to determine the cause of a change in impedance.

### 4.2. The Impact of Varying Counter Electrode Size on Electrochemical Behaviour

Any charge passed at a working electrode must be balanced by the opposite charge at the counter electrode. For a reduction current occurring at the working electrode, oxidation current must be passed at the counter electrode and vice versa. The current passing through the counter electrode will change its potential versus the reference electrode, but this can be ignored in a three-electrode configuration. A large counter electrode is used, so the majority of charge can be supplied by capacitance. When capacitance is insufficient, a redox reaction must occur on the counter electrode. This can lead to generation of gas or other chemical species, which can contaminate the solution, change the concentration of chemical species including pH, and block the electrode (e.g., formation of a bubble). To limit these interferences, the counter electrode should be kept a sufficient distance from the working electrode.

When a redox reaction occurs at the counter electrode (or working electrode), mass transport and electron transfer kinetics play a role. By using a large counter electrode capable of fast electron transfer and a large capacitance charge, the rate-limiting charge transfer steps (mass transport and electron transfer kinetics) are dominated by the working electrode. Reducing the counter-electrode size reduces its capacitance and increases its reliance on Faradaic current. This can lead to the counter electrode limiting the rate of charge transfer. An electrode material with slow electron transfer kinetics can enhance this effect. An electrochemical response in a cell with a small counter electrode can then display contributions from either or both working and counter electrodes, complicating or preventing any analysis, which is further discussed in the following section.

Another impact arises from changing electrode size and capacitance (*C*), which can be described by the Helmholtz model,

(2) $$C=\varepsilon \varepsilon_{0}A/d$$

where *ε* is the dielectric constant of the solution, *ε*_0_ is the permittivity of free space, *A* is the electrode area, and *d* is the thickness of the double layer. The total cell capacitance (*C*_T_) is controlled by the capacitance of the working electrode (*C*_WE_) and the counter electrode (*C*_CE_),

(3) $$C_{T}=C_{\mathrm{WE}}C_{\mathrm{CE}}/(C_{\mathrm{WE}}+C_{\mathrm{CE}})$$

When *C*_CE_ is ~30 times greater than *C*_WE_, *C*_T_ is dominated by the working electrode. Changes in *C*_WE_ due to electrode potential, composition, and size can then be measured. This is further enhanced by using the same counter electrode across experiments. By reducing the size of the counter electrode to an equivalent size of the working electrode, *C*_T_ is halved. Any variations in counter-electrode size and capacitance have a large impact on *C*_T_. This was seen with a decrease in CSC with decreasing counter-electrode size (Table 1). An increase in impedance at 10 Hz and decrease of admittance, consistent with decreasing *C*_T_ [31], were within error (Table 2). These values did not halve or double as some charge was associated with Faradaic current. Reducing the size of the counter electrode did not affect the Faradaic current from the Ru(NH_3_)_6_^3+^ voltammetry by limiting charge transfer kinetics (Figure 4a), but larger concentrations/currents may.

Use of a large counter electrode, placed far from the working electrode, generally leads to a uniform current density within the solution and across the electrode surfaces. However, a small counter electrode placed relatively close to the working electrode, as is often performed in bipolar electrode configurations, can create non-uniform current densities. In particular, high current densities may occur on working and counter-electrode edges when they are placed side-by-side. This can result in higher-than-expected potentials on some electrode regions, leading to non-uniform electrochemical reactions, such as corrosion. Modelling of individual electrode geometries and positions must be performed to determine the charge density distribution.

While uncompensated resistance occurs between the reference and working electrode, the majority of charge passes between working and counter electrodes. While a high resistance between these electrodes will not impact the electrochemical response, it will require more power. Power usage is not usually important for bench-top electrochemistry but is an important consideration for battery-powered implantable devices. To minimise power usage, the resistance between counter and working electrodes should be reduced by increasing solution conductivity or reducing the distance between them. Where possible, the amount of poorly conductive bone between the electrodes should be limited. Careful design of an electrode surface may allow tailoring of the power usage and charge transfer mechanisms occurring during stimulation of tissue. If charge injection requires a Faradaic reaction, then a low overpotential is desirable for reducing power usage. Conversely, if Faradaic reactions are undesirable (e.g., corrosion, formation of toxic species, or blocking the electrode), then slow kinetics would reduce their contribution to current flow.

### 4.3. The Impact of Using a Two-Electrode Configuration on Electrochemical Behaviour

Typically, electrochemical systems operate in a three-electrode configuration with the majority of current passing through the counter electrode, ensuring the reference potential is stable. In a two-electrode configuration, the counter and reference electrodes are combined. As a result, current passing through the counter/reference electrode alters its chemical state and potential. When the counter/reference electrode is sufficiently large and the current draw is small, the charge may be supplied by capacitance, with limited impact on its potential. As the electrode decreases in size and the current draw increases, more charge must be supplied by Faradaic reactions, with greater impact on its potential. For instance, in a two-electrode configuration with the large platinum mesh counter/reference electrode in 0.1 M NaCl, the voltammetry was relatively consistent over multiple cycles; however, increasing charge passage by the addition of 5 mM Ru(NH_3_)_6_^3+^, the *E*^1/2^ shifted 5–10 mV over subsequent cycles.

Another impact in using a two-electrode configuration is that the working-electrode potential is no longer defined versus a standardised reference potential. The potential is now defined as a potential difference between working and counter/reference electrodes. This potential difference can no longer be calibrated versus a standard reference potential. Defining a safe potential window of 0.8 to −0.8 V versus a reference potential is no longer relevant in a two-electrode configuration. With a large counter/reference electrode, with a relatively stable potential, the safe potential window was still ~1.6 V, but could shift in absolute potential (Figure 2). When the counter/reference electrode size was reduced, the safe potential range increased to ~3.2 V, as sweeping in a cathodic direction, the working electrode potential can shift negatively and the counter/reference-electrode potential can shift positively. This also resulted in a shift in the Ru(NH_3_)_6_^3+^ reduction peak to −1392 mV, ~900 mV more negative than in the three-electrode configuration. Variations in working and counter/reference-electrode size, structure, solution composition, etc., can subsequently impact on the safe potential range and Faradaic potentials. Partial blocking of the electrode surface by protein adsorption, reducing its effective area [13], can also change these potential values. It also leads to changes in the shape of Faradaic curves, such as an increase in peak width, which may prevent the use of common analytical methods and equations (e.g., Equation (1)).

The current definitions are also affected by the electrode configuration. In a three-electrode configuration, a cathodic Faradaic current is due to a reduction reaction occurring on the working electrode, and an anodic Faradaic current is an oxidation reaction. The reactions occurring on the counter electrode can be ignored. However, in a two-electrode configuration, current is measured through working and counter/reference electrodes. The shape of an electrochemical response is determined by the rate-limiting charge transfer step. So, what appears to be a cathodic Faradaic current can be caused by an oxidation reaction occurring on the counter/reference electrode (with capacitance occurring on the working electrode) and, conversely, an anodic Faradaic current can be due to a reduction reaction. Limitations from mass transport and electron transfer kinetics at working and counter/reference electrodes can make it very difficult to determine what the rate-limiting charge transfer steps are and what reactions are occurring at each electrode.

For instance, voltammetry of Ru(NH_3_)_6_^3+^ in a two-electrode configuration with similar-sized electrodes showed two irreversible Faradaic peaks (Figure 4b–d). The presence of an anodic peak at ~1420 mV without prior reduction of Ru(NH_3_)_6_^3+^ indicates that this peak is due to its reduction at the counter/reference electrode with a balance of capacitance and Faradaic charge being passed at the working electrode. The reduction of Ru(NH_3_)_6_^3+^ would alter the Nernst potential (due to the relative concentration of reduced and oxidised species) at the electrode. On switching the scan direction, while Ru(NH_3_)_6_^2+^ was now present at the counter/reference electrode, no oxidation peak occurred (as a diffusion-controlled cathodic peak). The charge transfer rate now appears to be limited by the working electrode. With the working electrode at this large positive potential, it can only supply charge by capacitance and possibly reduction of platinum oxide, and so the charge transfer rate is limited by the voltammetric scan rate. The oxidation of Ru(NH_3_)_6_^2+^ at the counter/reference electrode can only proceed at the same rate, preventing the formation of a current peak. Conversely, when the potential is approximately −1420 mV, reduction of Ru(NH_3_)_6_^3+^ can occur at the working electrode, but a similar effect occurs when switching the scan direction with oxidation of Ru(NH_3_)_6_^2+^ being prevented by rate-limiting charge transfer at the counter/reference electrode. There are also charge transfer rate limitations of the Ru(NH_3_)_6_^3+^ reduction caused by the other electrode, with the peak current being substantially smaller than in other electrode configurations. This has important ramifications for electrochemical sensing; if a calibration curve is created with a different electrode configuration, then measurements with a two-electrode configuration may be significantly undervalued. It also means that commercial electrochemical modelling software is no longer useful, as current limitations and potential variation of the counter electrode cannot be simulated.

Overall, use of a two-electrode configuration has impacts on several measured parameters, with a smaller counter/reference electrode having a larger effect. The CSC and charge density were smaller over a fixed potential range (Table 1). However, the CSC values can include contributions from both working and counter/reference electrodes, and the CSC over the entire safe potential window was larger. This larger CSC is also due to the increased measurement time over the wider potential window. The impedance at 10 Hz, solution resistance, and polarisation resistance increased while admittance decreased (Table 2). In addition, as mentioned previously, the Faradaic peak potentials shifted and peak currents decreased in magnitude.

### 4.4. Recommendations for Performing In Vivo Electrochemistry

Our previous work has demonstrated degassed saline is the best model solution for assessing in vivo electrochemistry [12]. However, minor changes in solution do not affect the overall conclusions from this article on varying electrode configuration. Given the impacts of electrode configuration discussed above, bench-top electrochemistry is normally performed in a three-electrode configuration with a defined reference electrode, large counter electrode, and a well-polished working electrode. Each experiment occurs over a relatively short time period (limiting reference potential drift), and the same electrodes are used for multiple experiments. However, this is generally not possible for in vivo electrochemistry. The same electrodes may be used for decades in a complex environment that blocks or modifies their surface; they cannot be cleaned after every measurement and their potentials can drift. It is common to use different electrodes and electrode configurations on an electrode array to stimulate or record local tissue function or chemical composition. The electrode configuration may be defined by anatomical, biological, or clinical outcomes and not for electrochemical analysis. In addition, the tissue/bone composition between electrodes can vary with configuration or over time, altering resistance and current flow.

The choice of electrode materials for in vivo use is limited by biocompatibility and biostability requirements [33]. Ag|AgCl cannot be used in long-term medical devices as Ag^+^ is toxic and AgCl is soluble. Platinum is the most commonly implanted electrode material, with surgical screws of titanium or stainless steel often used for counter or reference electrodes or an Ag|AgCl wire in saline-soaked cotton wool for acute experiments. Other electrode materials include iridium oxide, conductive organic materials, PtIr, nitinol, and gold, but the details of electrode materials, sizes, and electrochemical parameters are often not included in publications. Quite often, these materials and configurations will be changed between in vitro, acute, and chronic in vivo experiments and between different animal models or patients without calibration.

Further complications arise from the use of mono-polar or bi-polar stimulation. Mono-polar stimulation is defined as one electrode close to the target tissue with a counter electrode located some distance away so that current is forced through the surrounding tissue [34]. Bi-polar stimulation is defined as two similar sized electrodes close to the target tissue to limit current spread and off-target stimulation. The choice of electrode configuration is defined by the relevant electrophysiological or clinical outcomes. However, the same electrode configurations are also used to assess the electrode and tissue properties. In these measurements, it is not always clear if the electrodes are used in a three- or two-electrode configuration, as there is often a large electrode placed further away, some electrodes may be short-circuited, and the electronics may or may not be a potentiostat. Furthermore, if a distant, large electrode is short-circuited to a small bi-polar electrode to increase the total size of a ground electrode, high tissue resistivity may limit any current flowing through the larger electrode. Subsequently, it may not be clear which electrode is acting as a working electrode, the relative electrode sizes and locations, and the current flow. To overcome these issues, the type of electronics, electrode connections, configurations, sizes, locations, and materials must be clearly stated. Background measurements should also be performed to ensure the electrode configuration is not affecting any electrochemical analysis.

While electrode configuration can affect electrochemical behaviour, the degree of impact will depend on the experiment being undertaken. When performing electrophysiological recording, very low current is passed but, more importantly, the response is band-pass-filtered, removing the DC component. Subsequently, any issues of potential drift are removed. Electrode configuration effects would only be noticed if the counter electrode was extremely small or blocked, or the resistance extremely large. For electrophysiological stimulation (e.g., voltage transients), electrode configuration can have an impact, but it may not be important if only the electrophysiological or clinical function is measured. The biggest concern is when electrochemical analysis is being performed for electrode, tissue or electrochemical sensing. The level of impact will depend on numerous parameters, including electrode configuration, size, location, geometry, tissue composition, current draw, and electrochemical method. Some errors and the impact that may arise when altering the electrode configuration are listed below.

Calibrations performed in vitro may no longer be accurate when testing in vivo, which can lead to incorrect chemical measurements or assigned safety limits. Assessment of electrode function or tissue properties (e.g., through an impedance test using voltage transients) may no longer be valid [35]. When these measurements are used for clinical applications, they may result in incorrect diagnosis and clinical intervention, such as medication, leading to physical, mental, or social harms [36]. Devices may be used in an unsafe manner, leading to patient harm, or in an overly cautious manner, limiting patient benefit. It may be possible to correct for calibration errors by recalibrating in vivo or using a second measurement technique. However, these limitations have been recognized, with carbon fibres used for detecting neurotransmitters, including dopamine, only assessing relative changes in concentration and not absolute values, and concentration changes only made over relatively short time scales [37].

Reaction mechanisms determined from an electrochemical response in vitro may be altered or misinterpreted when performed in vivo. Similarly, analytical methods may no longer be valid with different electrode configurations. This may result in incorrect understanding of biochemical mechanisms.

Therefore, it is critical to know if an electrode configuration is affecting electrochemical behaviour and what steps can be taken to correct for it. An undefined and unstable reference electrode potential is expected for in vivo measurements. The stability of the electrode potentials should be assessed for individual devices and tissue locations to determine the impact on any experimental results. Its presence can be detected by changing OCP or shifting voltammetry, which may be sufficient for in vivo recalibration. However, changes in surrounding solution composition or adsorption of different species onto the electrode due to biological or electrochemical mechanisms may prevent use of this recalibration method. Addition of a redox species with a known concentration to calibrate the potential is not possible. A surface-confined redox process, such as platinum oxide reduction, may be available but the peak potential is not very accurate and may be affected by conditions. Subsequently, all in vivo electrochemical methods and analyses should take into account the expected variable reference potential, for instance, measures of peak potentials would be inaccurate, whereas peak area or height may be more stable.

In a three-electrode configuration, if the counter electrode is too small or its electron transfer rate is too slow, it can limit the charge transfer rate. This can be assessed by connecting a larger counter electrode and seeing if the current magnitude changes. So long as the counter electrode is sufficiently large and charge density is uniform, it will not affect any electrochemical analyses.

In a two-electrode system, if the counter/reference electrode is too small or its charge transfer rate is too slow, it can have significant impacts on the electrochemical response. It can be detected by an increase in potential window. Where a Faradaic response is present, changes in redox potential will also be observed. The impact can be reduced by using a larger counter/reference electrode or moving to a three-electrode configuration.

Overall, issues due to electrode configuration can be minimised by using a three-electrode system with a stable, defined reference electrode and large counter electrode with fast charge transfer kinetics. The electrodes should be close enough to minimise resistance but far enough away to prevent interference from chemical contamination, and changes in charge density (unless they are being used to direct current flow). When placed in a blood vessel, the reference electrode should be upstream of the working electrode, and the counter electrode should be downstream. Where the electrochemical response is affected by electrode configuration, the relative response may be assessed, but absolute measurements and speculation on its biological significance should be avoided.

## 5. Conclusions

The electrochemical responses of platinum electrodes were assessed in two- or three-electrode configurations with differing reference electrodes and counter-electrode sizes. Changes in electrode configuration affected the potential and current response and definitions. This manifested in different ways on open circuit potential, cyclic voltammetric and electrochemical impedance spectroscopic experiments. Use of a three-electrode configuration with a well-defined and stable reference electrode and large counter electrode enabled detailed analysis of the electrochemical behaviour at the working electrode. In a two-electrode configuration with similar working and reference/counter-electrode sizes, the electrochemical response was dictated by the rate-limiting charge transfer step at either electrode. This could invalidate calibration curves and standard analytical methods and equations. The electrode configuration used for in vivo electrochemistry and electrophysiology is often optimised for a specific anatomy and biological or clinical outcomes, not the electrochemical performance. Subsequently, in vivo electrochemistry can be impacted by changes in electrode configuration. The electrode configuration can also affect resistance and power usage, important parameters for battery-powered medical devices. To minimise errors from electrode configuration during electroanalysis, each system should be calibrated and the response assessed for variations with different reference electrode potential, counter-electrode size, and use of a two- or three-electrode configuration. Sufficient details should be provided in experimental sections on electrode configuration and calibration to justify any results and their discussion.

## Figures and Tables

**Table 1:** Open circuit potential, charge storage capacity, and charge density of a 0.6 mm diameter platinum electrode in different configurations over a potential range of 0.8 to −0.8 V at a scan rate of 100 mV s^−1^ *.

Electrode Configuration		Charge Storage Capacity/μC		Charge Density/mC cm^−2^	
	OCP/mV	Reduction	Oxidation	Reduction	Oxidation
3-electrode, Ag|AgCl RE	289 (10)	2.9 (0.3)	1.8 (0.2)	1.02 (0.09)	0.63 (0.07)
3-electrode, Ag|AgCl RE, Pt disc CE	236 (41)	2.2 (0.1)	1.4 (0.1)	0.77 (0.02)	0.51 (0.03)
3-electrode, Pt disc RE, Pt mesh CE	0 (5)	3.6 (0.2)	2.4 (0.1)	1.29 (0.08)	0.85 (0.03)
2-electrode, Pt mesh RE/CE	−38 (35)	1.9 (0.1)	2.0 (0.2)	0.68 (0.04)	0.72 (0.05)
2-electrode, Pt disc RE/CE	19 (3)	1.2 (0.1)	0.8 (0.1)	0.42 (0.02)	0.28 (0.01)

* Average (standard deviation) of 3 repeats.

**Table 2:** Impedance and equivalent circuit fitting parameters of a 0.6 mm diameter platinum electrode in different configurations *.

Electrode Configuration	Impedance 10 Hz/kOhm	*R*_1_/Ω	*Q*_0_/10^−8^ S s^1/2^	*n*	*R*_2_/MΩ
3-electrode, Ag|AgCl RE	95 (15)	1210 (9)	28.8 (4.6)	0.88 (0.01)	3.68 (1.78)
3-electrode, Ag|AgCl RE, Pt disc CE	110 (10)	1050 (150)	24.6 (2.4)	0.88 (0.00)	4.78 (0.43)
3-electrode, Pt disc RE, Pt mesh CE	135 (17)	1180 (9)	21.3 (1.5)	0.86 (0.01)	20.7 (4.48)
2-electrode, Pt mesh RE/CE	145 (13)	1230 (20)	20.1 (1.3)	0.86 (0.01)	36.9 (1.52)
2-electrode, Pt disc RE/CE	278 (9)	2210 (16)	12.3 (0.4)	0.84 (0.01)	26.0 (0.86)

* Average (standard deviation) of 3 repeats.

## Data Availability

Not applicable.
